# Social support and prosocial behavior in Chinese college students during the COVID-19 outbreak: a moderated mediation model of positive affect and parental care

**DOI:** 10.3389/fpsyg.2023.1127194

**Published:** 2023-05-10

**Authors:** Ziyang Huang, Quan Gan, Miaoling Luo, Yunpeng Zhang, Jie Ge, Yu Fu, Zhuangfei Chen

**Affiliations:** ^1^Medical School, Kunming University of Science and Technology, Kunming, China; ^2^Brain Science and Visual Cognition Research Center, Medical School of Kunming University of Science and Technology, Kunming, China; ^3^Faculté de Médecine, Université Paris-Saclay, Le Kremlin-Bicêtre, France; ^4^Faculty of Education, Yunnan Normal University, Kunming, China; ^5^Students Counseling and Mental Health Center, Kunming University of Science and Technology, Kunming, China

**Keywords:** social support, prosocial behavior, moderated mediation model, parental care, COVID-19

## Abstract

**Purpose:**

Prosocial behavior (PSB) plays a critical role in everyday society, especially during the pandemic of COVID-19. Understanding the underlying mechanism will provide insight and advance its implementation. According to the theory of PSB, social interaction, family and individual characters all contribute to its development. The current study aimed to investigate the influencing factor of PSB among Chinese college students during COVID-19 outbreak. This is an attempt to understand the mechanism of PSB and to provide a reference for the formulation of policies aimed at promoting healthy collaborative relationships for college students.

**Method:**

The online questionnaire was administered to 664 college students from 29 provinces of China via Credamo platform. There were 332 medical students and 332 non-medical students aged between 18 and 25 included for final study. The mediating role of positive emotion/affect (PA) and the moderating role of parental care in the association between social support and PSB during the pandemic of COVID-19 was explored by using Social Support Rate Scale (SSRS), Prosocial Tendencies Measurement Scale (PTM), The Positive and Negative Affect (PANAS), as well as Parental Bonding Instrument (PBI). The process macro model of SPSS was adopted for mediating and moderating analysis.

**Results:**

The results showed that social support positively predicted PSB among Chinese college students, even after adding PA as a mediation variable. PA during COVID-19 mediated the association between social support and PSB. PSB also revealed as a predictor of PA by regression analysis. Moreover, the moderating effect of parental care in the relationship between PA and PSB was detected.

**Conclusion:**

PA under stress acts as a mediator between social support and PSB. This mediating effect was moderated by PC in childhood. In addition, PSB was observed to predict PA reversely. The promoting factors and path between the variables of PSB are complex and need to be explored extensively. The underlying factors and process should be further investigated for the development of intervention plans.

## Introduction

The COVID-19 pandemic is a serious global public health emergency (Word Health Organization, 2020). The pandemic created a huge crisis for individuals, triggering stress responses both physically and psychologically (Tang et al., 2021). There is a growing need for the public to be involved voluntarily in the prevention work (Gallaway et al., 2020). It has been pointed out that the effective implementation of policies for controlling the pandemic is largely dependent upon compliance from the public (Han et al., 2023). The pandemic situation brought collective pressure and a rapid switch in daily life. Face-to-face communications have been greatly reduced, accompanied by negative emotions. The pandemic has also brought about major challenges to the implementation of disease prevention policies in public health. Supportive interactions were proven to show benefits in promoting public compliance, even only via daily efforts such as frequent hand washing, wearing masks and maintaining social distancing (Alzyood et al., 2020; Howard et al., 2021; Deng and Chen, 2022). These public measures, and interpersonal support, can contribute to PSB when it relates to social expectations and benefit other groups and society (Huang, 2004). This can be an effective indicator for individual mental health (Son and Padilla-Walker, 2020; Alvis et al., 2022) and moral development (Eisenberg et al., 2002b), encouraging individuals to adapt effectively to society and secular moral standards.

Prosocial behavior is considered a positive reaction mode gradually developed in the process of integrating self and the external world. The cultivation of PSB in young adults is of great significance to their individual psychological development (Yang et al., 2015). It has been demonstrated that adolescents present more helping behaviors during critical situations, including in the COVID-19 pandemic (Liu et al., 2021; Alvis et al., 2022; Sweijen et al., 2022). Though the pace of life and learning habits were greatly affected, college students still presented more helping behaviors (Rotenberg et al., 2005) in this period. It is worthwhile to explore the underlying mechanisms of PSB in college students. Furthermore, it should be taken into consideration how psychological and external environmental factors initialise and stimulate helping behaviors, and whether prosocial behavior in turn promotes emotional health (Liu et al., 2021).

Although, the reasons for “helping” conduct become increasingly complicated with age. The PSB for teenagers may reflect their enhanced sense of social moral responsibility (Carlo et al., 2003; Brandenberger, 2005). It has been proposed that PSB originates from the internalization of social ethics in the early years of life (Helena and Bernardes, 1997; Hardy and Carlo, 2005). The research on children and adolescents also found that PSB showed longitudinal continuity in different stages of development (Luengo Kanacri et al., 2014). It was suggested that paying attention to prosocial behavior education in childhood, is helpful to the development of moral personality (Winer, 2013), in terms of the cultivation of specific prosocial behaviors, that have been gradually integrated into family and school education (Reiman, 2009). The concept of PSB education in other cultural backgrounds has confirmed this observation (Yu, 2010). At the same time, according to the motivation theory (Gan and Zuo, 2006), PSB is also related to the activation of appropriate external environmental factors under internalized moral norms. In emergency situations, emotional factors play a major role in the decision-making process of PSB. It has been pointed out that a positive mood promotes prosocial behavior (Kou and Tang, 2004).

According to the information processing model of social adaptation (Crick and Dodge, 1994), the connection between intention and behavior of PSB contact was affected by individual abilities and needed to be promoted. On the other hand, social learning theory indicates that individual behavior characteristics are mainly affected by the social environment (Bandura, 1977). College students are at the stage of rapid changes in psychological development and are immersed in more social information beyond family and school. They have a greater desire to willingly participate in peer interaction and are inclined to show more helpful behavior (Yang et al., 2020).

Generally speaking, theories about PSB primarily emphasize the interaction of social, family and individual characteristics. The perspective of social support, an individual’s emotional state and family-rearing history, should be taken into consideration (Zhang, 2017). With a decrease in interpersonal communication during the pandemic, it is worthwhile to explore the underlying mode of cultivation of PSB. The promotion of PSB can be discussed from the cross-sectional and longitudinal dimensions, as well as the internal and external factors of individuals. Besides, it would strengthen the theoretical basis for the cultivation of PSB in young adults (China Central Television (CCTV), 2021).

### Social support and prosocial behavior

Social support refers to the tangible and intangible resources people obtain at the individual, group and organizational levels through social interactions. Social support theory highlights the prosocial facets of human relationships and the support provided by an individual’s social environment. It expands beyond the individuals to their families, and communities, and aims to think of alternative social security-building policies (Chouhy, 2019). People with greater social capital (i.e., resources and benefits gained from relationships, experiences, and social interactions) may be more likely to be prosocial bystanders (Jenkins and Fredrick, 2017). Individuals with higher levels of social support showed more PSB (Gest et al., 2010). Active social support provides a good environment for the practice and development of PSB (De Guzman et al., 2012).

During the pandemic, it was reported that social support was increasing in Chinese adolescents (Kuang et al., 2020; Geng et al., 2022). Moreover, it can positively predict PSB (Kou and Zhang, 2006; Wang, 2011). College students presented with more PSB tendencies though facing the change of learning style and the decrease in interpersonal communication. It is worthwhile to further reveal the role social support plays during the process (Liu et al., 2021).

Herein, the current study assumes that social support can positively predict PSB among Chinese college students during the pandemic. Thus, we proposed that:

*Hypothesis 1*: Social support can positively predict prosocial behavior among Chinese college students.

### The mediating role of positive affect during COVID-19

The intention of PSB or helping behavior was largely induced in critical situations via internal motivation factors (Zheng et al., 2021). The temporary positive emotional state, positive interpersonal experience (Cirelli et al., 2014; Li et al., 2019) as well as active social support will further promote the underlying process (Eisenberg et al., 2002a).

According to Barbara Fredrickson’s expansion-construction theory (Isgett and Fredrickson, 2015), positive emotion is conducive to expanding the scope of individual attention, cognition and action, and enhancing social flexibility. It will further encourage individuals to actively participate in interpersonal activities, building long-lasting resources (Chen et al., 2017; Li et al., 2019) and more likely to show PSB (Fredrickson, 2004). Furthermore, in interpersonal help situations, individuals tended to identify with the just behavior of others (Michie, 2009), and also to follow the role models (Greitemeyer, 2022). Accordingly, public implementation of PSB can enhance well-being (Miles et al., 2021) and promote positive emotion (Varma et al., 2023), even among volunteers (Li, 2020). Though heterogeneous in adult samples, more positive emotion and well-being were reported in college students during COVID-19 pandemic. Besides, it was suggested there is a link between prosocial behaviors and positive emotions (Snippe et al., 2017). From the perspective of pandemic prevention and control, this study will pay more attention to the combined mediating effects of positive emotion, social support, and PSB. It is necessary to further clarify the indirect effect of positive emotion on prosocial behavior tendency. To better understand the possible role that positive emotion plays in the promotion of PSB, we put forward the second hypothesis:

*Hypothesis 2*: Positive affect during COVID-19 would play a mediating role between social support and prosocial behavior.

### The moderating role of parental care

It has also been proposed that the inheritance of family culture, in particular family education factors, can affect the development of PSB tendency. According to the internalization model, children and adolescents develop expectancies regarding their parents’ reactions to their behaviors. To be specific, positive parenting in the form of parental care and guidance can reinforce parents’ values of care and respect for others (Grusec and Goodnow, 1994) through exemplary role models (Augustine and Stifter, 2015). It also contributes to promoting the development of children’s social abilities (Caplan et al., 2019), greatly affecting the formation of secure attachment (Liu et al., 2009). Mother’s rearing style was revealed to be related to PSB in children. In experimental conditions, improving attachment security can enhance self-transcendence, thus promoting the occurrence of PSB (Wu, 2009). Retrospective studies also pointed out that early attachment security in adolescents can predict PSB during the COVID-19 pandemic (Coulombe and Yates, 2021). Therefore, this study hypothesized that early parental care played a moderating role in positive emotion and PSB during the pandemic.

*Hypothesis 3*: Parental care may moderate the relationship between positive affect (PA) during COVID-19 and prosocial behavior.

**Figure 1:**
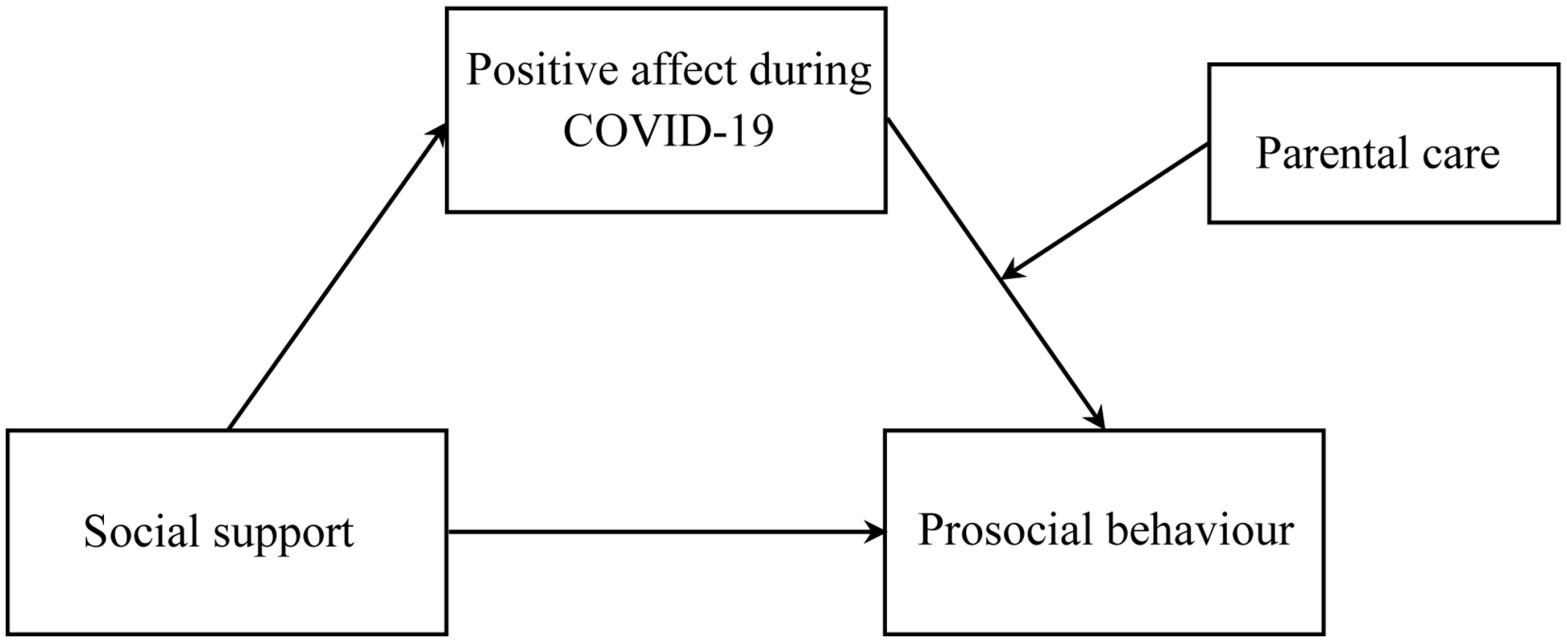
The proposed moderated mediation model.

As reviewed above, we constructed a moderated mediation model to examine the medial effect of positive affect (PA) during the COVID-19 pandemic. We further expected the indirect path between social support and prosocial behavior would be moderated by parental care in childhood. The theoretical model was presented in Figure 1.

## Methods

### Participants and procedures

Credamo is an online professional data platform for research use with over 3 million pooling participants. It has provided research and data services for more than 2,000 universities and scholars around the world (Credamo, 2023). The studies accomplished by using Credamo, have been published in international top journals in the fields of psychology, management, sociology, and public health.

In this online survey, Credamo randomly distributed questionnaires in 29 provinces of China. The survey ran from February 8 to February 16, 2021, 18- to 25-year-old undergraduates were randomly invited to participate in the study. There were 731 responders completed the task. The researcher assured all participants voluntarily involved and their anonymity, confidentiality, and the information every respondent provided was only for study purposes. Screening questions were set up in Credamo in advance to ensure the authenticity of the data. Those who failed the examination questions (e.g., answering the wrong “please choose very satisfied” instruction) and with inconsistent or contradictory answers were rejected. Finally, with 67 participants excluded, the actual number of valid questionnaires was 664. Among those participants, 332 medical students and 332 non-medical students were included. The final sample consisted of 52 freshmen (7.8%), 164 sophomores (24.7%), 204 juniors (30.7%), and 244 seniors (36.75%, 194 in grade 4 and 50 in grade 5), including 247 males (37.2%) and 417 females (62.8%). The participants all had normal visual acuity and no mental illness. The study was approved by the Medical Ethics Committee, Kunming University of Science and Technology (approval number: KMUST-MEC-142).

### Measures

#### Social support

Social support was assessed by using the Social Support Rate Scale (SSRS; Xiao, 1994). The scale is a self-administered questionnaire originally developed by Chinese scholar. At present, it has been mainly used to measure the social support of the general population, patients and medical staff in Chinese population (He et al., 2008; Chen et al., 2014). The scale consists of three dimensions, including subjective support, objective support and utilization of support. The total score of three dimensions was used to evaluate social support, with a higher score indicating stronger social support. It presented good reliability and validity in Chinese college students (Zhang, 2007). In this study, Cronbach’s alpha value for this overall scale was 0.748. The alpha coefficients for the three subscales were 0.637, 0.510, and 0.586. The reliability and validity were similar to that reported in a recent study among Chinese college students (Lu et al., 2023).

#### Prosocial behavior

The Prosocial Tendencies Measurement Scale (PTM) was developed by Carlo (Carlo and Randall, 2002). The Chinese version (Kou et al., 2007) was used to assess PSB in the participants. The PTM is a 5-point Likert scale, consisting of 23 items, broadly used in Chinese college students with good reliability and validity (Wang et al., 2021). A higher total score indicated a higher PSB tendency. It was categorized into six dimensions, including openness, anonymity, altruism, compliance, emotion and urgency. The Cronbach’s alpha was 0.866 for this overall scale. The alpha coefficients for each dimension were as follows: openness (0.742), anonymity (0.809), altruism (0.578), compliance (0.755), emotion (0.648), and urgency (0.592) in the current study.

#### The positive and negative affect

The PANAS scale was developed by Watson et al. (1988). The Chinese version (Huang et al., 2003) was developed based on the original one, which consists of 20 items, with each of 10 items evaluating positive and negative effects. The Cronbach’s alpha was 0.82 for the whole scale, that for Positive Affect and Negative Affect subscale were 0.85 and 0.83, respectively. The Likert 5-point scoring is used to measure the affection during the COVID-19 outbreak for participating college students. Participants rated all items from 1 (not at all) to 5 (very high). The higher the score, the more positive or negative emotions the individual experiences. PANAS was proved to be validated in China during COVID-19 (Tian et al., 2021). For the present study, the Chinese version of the scale was used, employing only the 10 items from the Positive Affect factor. The internal consistency (Cronbach’s alpha) for the sample in this study was 0.892.

#### Parental bonding instrument

PBI is a retrospective self-report questionnaire based on attachment theory developed by Parker (Parker et al., 1979), aiming to measure care and protection for each parent. The Chinese version (Yang et al., 2009) of the scale is composed of two parts, the Mother version (PBI-M) and the Father version (PBI-F) both containing 23 items. There are three subscales for each part, i.e., caring, encouraging autonomy, and control. In this study, the scale was used to measure only the part of the father and mother’s care. The total score of the father and mother’s care was considered as a measurement of parental care. The scale was proved to be of satisfied validities and variabilities and suitable for Chinese undergraduates (Yang et al., 2009). The higher the score, the stronger the subjects’ feeling of family warmth. In this study, Cronbach’s alpha for maternal care, paternal care and parental care were 0.798, 0.795, and 0.844.

### Statistical analysis

In the first place, the descriptive information of variables was analyzed, and the Pearson correlation coefficient was calculated to examine the relationships between these variables. Secondly, the process macro model 4 (a simple mediating model; Hayes, 2013) of SPSS 22.0 was used to test the moderating properties of positive affect (PA) during the COVID-19 outbreak, between social support and prosocial behavior. Next, the process macro model 14 of SPSS was used to investigate the moderating effect of parental care on the mediation effect, that is, whether the prediction of positive emotion on PSB was regulated by parental care. In the model, all continuous variables were normalized to be more comparable. A bootstrap test was conducted and the resultant 95% confidence interval was inspected to determine the significance of the results for mediating and moderating analysis. Confidence intervals without zero indicated significant impact. Additionally, unary linear regression mode was used to further reveal the prediction effect of PSB on PA reversely.

### Control and testing of common method biases

Before the data analysis, we adopted several methods to reduce common method deviations, including anonymity of participants, rearrangement and reverse expression of questions. The Harman one-way factor analysis was used to test for common method biases. There were 20 factors with eigenvalues >1 when unrotated, which explains 62.976% of the variance. The amount of variance explained by the first common factor was 16.951%, less than the critical value of 40%. Thus, no serious common method bias was detected in the current study.

## Results and analysis

### Descriptive and correlation results

In the current study, medical and nonmedical students were recruited. Descriptive information of the variables according to major was provided in Table 1. The results showed medical students reported a more positive affect (PA) during COVID-19 (*t* = 3.09, *p* < 0.01), while no significant difference was observed in social support, PSB and parental care (*p* > 0.05).

**Table 1:** Descriptive information of the variables in the medical students and non-medical student groups.

	Major	*N*	*M (SD)*	*t*	*p*
SS	MS	332	37.45 (6.56)	1.58	0.11
	NMS	332	36.67 (6.08)		
PA	MS	332	32.66 (6.40)	3.09	0.00
	NMS	332	31.05 (7.05)		
PSB	MS	332	81.30 (10.75)	1.72	0.09
	NMS	332	79.93 (9.62)		
PC	MS	332	41.79 (6.97)	1.58	0.12
	NMS	332	40.86 (7.62)		

SS, social support; PA, positive affect; PSB, prosocial behavior; PC, parental care; MS, medical students; NMS, non-medical students.

In correlation analysis, positive correlation effects were detected between any two of the variables. To be specific, PSB was positively related to social support (*r* = 0.38, *p* < 0.01), positive affect during COVID-19 (*r* = 0.43, *p* < 0.01) as well as parental care (*r* = 0.30, *p* < 0.01). Moreover, positive affect during COVID-19 was positively related to social support (*r* = 0.52, *p* < 0.01) and parental care (*r* = 0.45, *p* < 0.01; Table 2).

**Table 2:** Descriptive statistics and correlations for all variables.

	*M*	*SD*	1	2	3	4
1. SS	37.06	6.33	1			
2. PA	31.85	6.78	0.52**	1		
3. PSB	80.61	10.22	0.38**	0.43**	1	
4. PC	41.39	8.31	0.42**	0.45**	0.30**	1

*N* = 664. SS, social support; PA, positive affect; PSB, prosocial behavior; PC, parental care;**p < 0.01.

### The mediating role of positive affect

The mediating role of positive affect (PA) in the relationship between social support and PSB was tested by utilizing Model 4 of the PROCESS macro. As shown in Table 3, social support positively predicted PSB (*b* = 0.62, *t* = 10.63, *p* < 0.001). After adding PA as a mediation variable, social support was still a significant predictor of PSB (*b* = 0.35, *t* = 5.35, *p* < 0.001). Moreover, social support positively predicted PA (*b* = 0.56, *t* = 15.72, *p* < 0.001), and positively predicted PSB (*b* = 0.48, *t* = 7.93, *p* < 0.001). Furthermore, the bootstrap test indicated the significant indirect positive (CI = 0.19 to 0.36, effect size = 0.27) in Table 4, the mediation effect accounted for 43.55% of the total effect. Positive affect (PA) partially mediated the relationship between social support and PSB. The reverse was seen in the unary linear regression analysis, PSB also predicted PA during the pandemic of COVID-19 among university students (*B* = 8.788, SE = 0.286, *t* = 12.305, *β* = 0.431, *p* < 0.01).

**Table 3:** Testing the mediation model of positive affect (during COVID-19).

	Model 1		Model 2		Model 3	
Predictors	PSB		PA		PSB	
	*b*	*t*	*b*	*t*	*b*	*t*
PA					0.48	7.93***
SS	0.62	10.63***	0.56	15.72***	0.35	5.35***
*R^2^*	0.15		0.27		0.22	
*F*	112.99***		247.20***		93.18***	

*N* = 664. SS, social support; PA, positive affect; PSB, prosocial behavior; ***p < 0.001.

**Table 4:** Decomposition table of the total effect, direct effect, and mediating effect.

	Effect	*Boot S.E.*	*95%CI*	
			Lower	Upper
Total effect	0.62	0.06	0.50	0.73
Direct effect	0.35	0.07	0.22	0.48
Indirect effect	0.27	0.04	0.19	0.36

### The moderated mediation model

To examine whether parental care would moderate the indirect relationship between social support and PSB through positive affect (PA). The PROCESS macro (Model 14) was used to test the moderated mediation mode. In detail, the moderating effect of parental care on the relationship between social support and PSB was estimated (Table 5). Model 2 indicated that there was a significant main effect of positive influence on PSB, *b* = 0.44, *t* = 6.99, *p* < 0.001. The results of the test indicated this was moderated by parental care (*b* = 0.02, *t* = 4.24, *p* < 0.001).

**Table 5:** Moderated mediation effects of parental care on the relationship between positive affect during COVID-19 and prosocial behavior.

	Model 1		Model 2	
Predictors	PA		PSB	
	*b*	*t*	*b*	*t*
PC			0.13	2.69**
PA			0.44	6.99***
PA × PC			0.02	4.24***
SS	0.56	15.72***	0.31	4.70***
*R^2^*	0.27		0.25	
*F*	247.20***		53.82***	

*N* = 664. SS, social support; PA, positive affect; PSB, prosocial behavior; PC, parental care.

**p < 0.01, ***p < 0.001.

**Figure 2:**
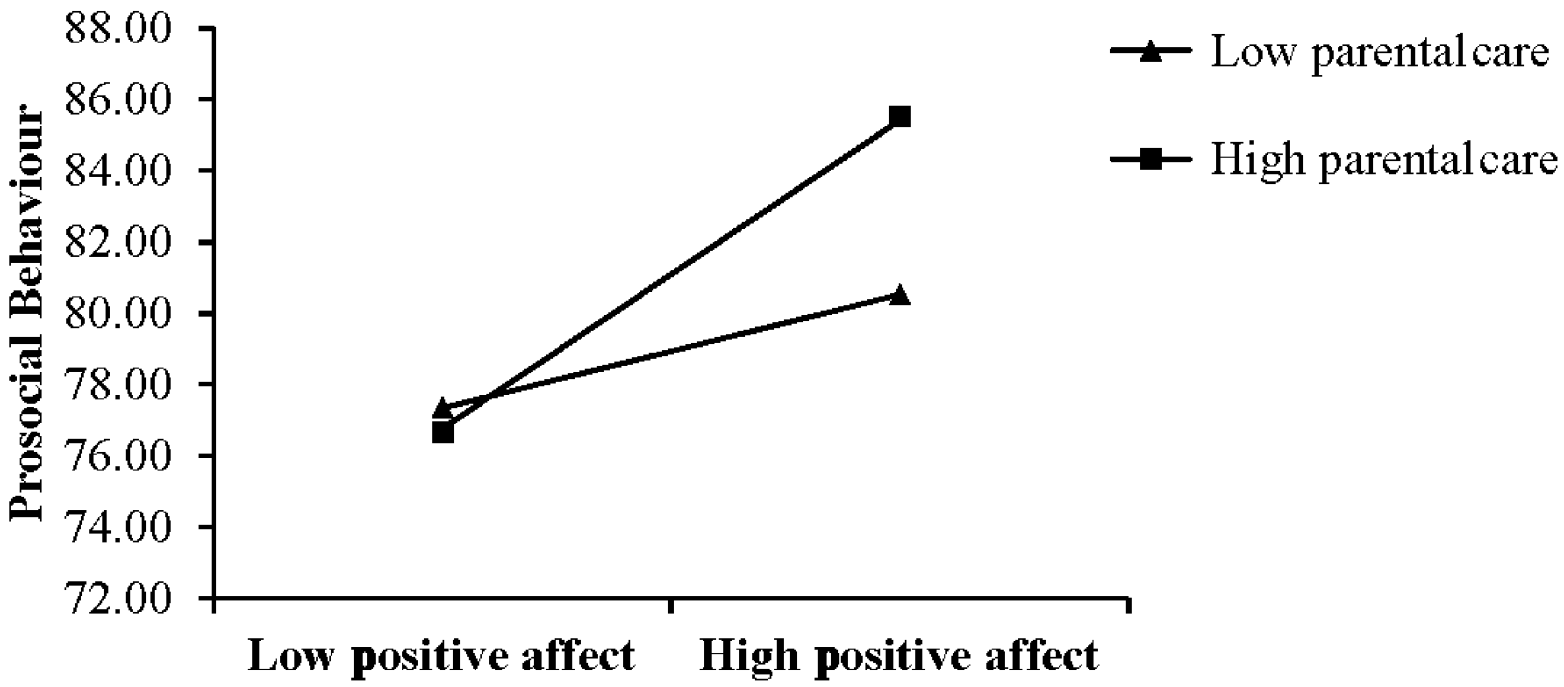
Parental care moderated the relationship between positive emotion during COVID-19 and prosocial behavior.

Table 6 shows the results of the bootstrap test, which revealed the conditional indirect effect of parental care. The 95% confidence intervals did not include a 0. In other words, the moderating effect of parental care through PA on PSB differed for individuals who reported low (i.e., M–1SD), average (i.e., mean), and high (i.e., M + 1SD) levels of parental care. Positive affect (PA) could positively predict PSB during the COVID-19 period among individuals with lower parental care (M–1SD, simple slope = 0.24, *t* = 2.97, *p* = 0.003). The mediated effect value elevated to 0.3 among individuals with higher parental care (M + 1SD, simple slope = 0. 64, *t* = 8.18, *p* < 0.001), indicating an enhanced prediction. The results suggested that, during the COVID-19 outbreak, the prediction effect of positive affect (PA) to PSB increased along with parental care level in childhood (Figure 2).

**Table 6:** Conditional indirect effect of parental care when positive affect during COVID-19 mediated between social support and prosocial behavior.

Mediator	PC	Estimate	S.E	95%Cl	
				Lower	Upper
PA	M-1SD	0.13	0.05	0.04	0.23
	M	0.24	0.04	0.17	0.33
	M + 1SD	0.36	0.26	0.26	0.47

*N* = 664. PA, positive affect; PC, parental care.

## Discussion

A variety of studies have tried to elaborate on the relationship between social support and PSB. Many of the impact factors were referred. In the current study, positive emotion as a situational (Eisenberg et al., 2002a) element has been verified to cast a mediation effect on the process. The present study further examined the moderating effect of parental care. Results showed that positive emotion during COVID-19 played a mediating role between social support and PSB, and this mediating effect was moderated by parental care in childhood among university students.

### The relationship between social support and prosocial behavior in college students

First, in line with previous studies on Chinese students, the contribution of social support to PSB (Li, 2010) was observed. In the context of the pandemic, the promotion of PSB may contribute to the setting of prosocial role models through social media (Greitemeyer, 2022), the workshops or open classes of preventative knowledge, as timely social support that strengthened a sense of concern and protection from social communities (Wouter et al., 2018; Kislyakov and Shmeleva, 2021).

According to the buffer model, social support is an effective intervention for individuals to deal with stressful events. Higher levels of social support among college students correlated to a more active, seeking mindset, in dealing with negative emotions (Liu, 2016). Emotion regulation and self-efficacy were improved for individuals where social support is concerned (Zeng and Suqun., 2012). Individuals with higher social support were more likely and tended to get help and guidance in time, also making it possible to resonate with others (Wei, 2020). The occurrence of PSB would be a consequence of improving physical and mental health (Liu et al., 2016).

Consistent with previous studies, and the extended exploration of critical situations, our results suggest that college students with high social support showed a higher tendency to PSB during the pandemic. To summarize, in the critical situations of the pandemic, social support played a positive role in promoting PSB in the buffering of stress (Mcguire et al., 2020; Chen et al., 2021), role model demonstration (Feng, 2016), positive publicity (Hu, 2020) and so on.

### Mediating effect of positive affect

In the current study, it was demonstrated that positive emotion partially mediates the relationship between social support and PSB during the COVID-19 outbreak. As reviewed above, people are more mindful to pay attention to the needs of others when in a positive emotional state, which also shows PSB in the context of help-seeking (Yu, 2010). The intention of PSB was largely created in critical situations through empathy (Eyal et al., 2018), which was further enhanced in temporary positive emotional states (Eisenberg et al., 2002a). Prosocial behavior is more likely to be aroused by the sharing of success and joy in helping others (Tan et al., 2020). At the same time, social support, as a factor of protection and also acted as a buffer during stress promoting positive emotions (Wang et al., 2022; Jasiński and Derbis, 2023) and subjective well-being (Yıldırım and Celik, 2020), thus possibly elevating the occurrence of PSB (Snippe et al., 2017). Previous studies also reported that the cultivation of PSB in children during the pandemic contributed to the shaping of mental health and resilience (Coyne et al., 2021). The mutual predicting effect between PSB and positive emotion (Snippe et al., 2017) was also detected in the current study. Moreover, medical students reported more positive effects during COVID-19 in the current investigation. This was in line with previous research of medical student volunteers (Zhang et al., 2021) and health workers (Mo et al., 2021). Most students reported being able to maintain a positive mindset during the COVID-19 pandemic (Zhao et al., 2021). However, there was still a transition in the emotional state of volunteers over time (Zhang et al., 2021). A longitudinal design is probably needed to detect the causation of major and emotional states under stress.

### Moderating effect of parental care

In this study, it was observed that parental care casts a positive predictive effect on PSB. The interaction of positive emotion and parental care was also probed, suggesting that the mediating effect of positive emotion on PSB is reinforced by parental care. Previous studies on parental rearing styles and PSB are mostly focused on children, adolescents or young adults (Mestre et al., 2007; Han, 2018). But there are few retrospective studies on the related factors of PSB in critical situations (Petrocchi et al., 2021).

According to the attachment theory, individuals who are safely attached in their early years are more likely to form interpersonal connections (Tuo, 2014). Those children tended to establish interpersonal relationships actively, showed higher social support, and an ease of participation in prosocial activities (Su, 2019; Zhang, 2019). The moderate rearing style plays a positive role in promoting PSB (Mestre et al., 2007). In our study, parental care showed a moderating effect on the process of positive emotion and PSB. The result of the moderated mediation model indicates that more social attention is needed in this population to create more positive emotions, especially during the pandemic. It can be a possible way to promote the occurrence of their prosocial behavior, emphasizing the importance of childhood parental care in individual growth. Changes in the external environment did act as a trigger factor in the process of promoting PSB (Figure 2). In short, parental care in childhood is of vital significance to PSB in critical situations in college students.

To summarize, mainly based on the theory of PSB (Rosser, 1981), broaden-and-build theory (Isgett and Fredrickson, 2015) and attachment theory (Schroeder et al., 1995), this study discussed the mediating effect of social support on PSB. The moderating effect of parental care was also examined. Our results suggest that positive emotions in critical situations play an incomplete intermediary role between social support and PSB. Parental care positively regulates the mediating process. There is an interaction between positive emotion and parental care in childhood, which suggests that parental rearing in early childhood can better promote PSB. It provides new clues to understanding the promotion of PSB and the significance of the process. Namely, strengthening social support in college student is beneficial for promoting prosocial behavior tendency. The temporary positive affect during stress also serves as a target for its mediating role in the procedure. The promotion of PSB can enhance individuals’ positive emotions in turn. Early family education can moderate this mediation process. Therefore, the promotion of PSB may need to be approached from multiple aspects, including current social support, personal emotional state, and early family education, as these efforts reflect the ultimate aim of promoting mental health in adolescent and young adults.

### Limitations and future direction

First of all, according to social learning theory, the development of PSB is an interactive process of cognition, behavior and environment. Although a cross-sectional survey was adopted in the present study, the relationship between variables still needs to be observed dynamically. Detection at a single time point is not sufficient to support a comprehensive understanding of the processes. Moreover, in the current study, the measurement of parental care relies on retrospective self-reporting, which may possibly be influenced by inaccurate memory. Finally, although this study discusses the mediating role of positive emotion and parenting, the promoting factors and path between the variables of PSB are still extremely complex and need to be explored more extensively. In addition, we only examined the model in the total sample due to the difference not being significant for each subjective measurement dimension between medical and non-medical students. The impact of majoring in mediating the effect of positive affect is still worth exploring in future studies.

Therefore, a time-event analysis (Ramseyer et al., 2014) is probably needed in the future work. Experimental and longitudinal studies should be included to further investigate the causal relationship of variables. The PSB events also need to be clearly defined and stratified by degree of effort. Prospective study (Malamut et al., 2021) was recommended regarding the interaction between individual, parental rearing, and or social environment (e.g., community/school). The underlying protective and risk factors, as well as resources (Alshehri et al., 2020; Arslan and Yıldırım, 2021a,b; Yıldırım et al., 2022) should be further explored, in order to facilitate the development of more concrete intervention plans.

## Data availability statement

The raw data supporting the conclusions of this article will be made available by the authors, without undue reservation.

## Ethics statement

The studies involving human participants were reviewed and approved by the Medical Ethics Committee, Kunming University of Science and Technology (approval number: KMUST-MEC-142). Written informed consent for participation was not required for this study in accordance with the national legislation and the institutional requirements.

## Author contributions

ZH and QG contributed to data collection, data processing, data analysis, statistical analysis, original manuscript drafting, and manuscript editing. ML, YZ, and JG contributed to data collection and data analysis. YF and ZC contributed to project conception, research design, and manuscript revision. All authors contributed to the article and approved the submitted version.

## Funding

This study was supported by the National Natural Science Foundation of China (NSFC; Nos. 32060196, 82201597, 31760281, and 81760258), Yunnan Ten Thousand Talents Plan Young and Elite Talents Project (YNWR-QNBJ-2018-027) and YN College Students’ innovation and entrepreneurship training program (S202210674097).

## Conflict of interest

The authors declare that the research was conducted in the absence of any commercial or financial relationships that could be construed as a potential conflict of interest.

## Publisher’s note

All claims expressed in this article are solely those of the authors and do not necessarily represent those of their affiliated organizations, or those of the publisher, the editors and the reviewers. Any product that may be evaluated in this article, or claim that may be made by its manufacturer, is not guaranteed or endorsed by the publisher.
